# Management of Protected Areas and Its Effect on an Ecosystem Function: Removal of *Prosopis flexuosa* Seeds by Mammals in Argentinian Drylands

**DOI:** 10.1371/journal.pone.0162551

**Published:** 2016-09-21

**Authors:** Claudia M. Campos, Valeria E. Campos, Florencia Miguel, Mónica I. Cona

**Affiliations:** 1 Instituto Argentino de Investigaciones de las Zonas Áridas (UNCuyo- Gobierno de Mendoza-CONICET), Mendoza, Argentina; 2 Centro de Investigaciones de la Geosfera y la Biosfera (UNSJ- CONICET), Interacciones Biológicas del Desierto (INTERBIODES), San Juan, Argentina; Sichuan University, CHINA

## Abstract

The ecological function of animal seed dispersal depends on species interactions and can be affected by drivers such as the management interventions applied to protected areas. This study was conducted in two protected areas in the Monte Desert: a fenced reserve with grazing exclusion and absence of large native mammals (the Man and Biosphere Ñacuñán Reserve; FR) and an unfenced reserve with low densities of large native and domestic animals (Ischigualasto Park; UFR). The study focuses on *Prosopis flexuosa* seed removal by different functional mammal groups: “seed predators”, “scatter-hoarders”, and “opportunistic frugivores”. Under both interventions, the relative contribution to seed removal by different functional mammal groups was assessed, as well as how these groups respond to habitat heterogeneity (i.e. vegetation structure) at different spatial scales. Camera traps were used to identify mammal species removing *P*. *flexuosa* seeds and to quantify seed removal; remote sensing data helped analyze habitat heterogeneity. In the FR, the major fruit removers were a seed predator (*Graomys griseoflavus*) and a scatter-hoarder (*Microcavia asutralis*). In the UFR, the main seed removers were the opportunistic frugivores (*Lycalopex griseus* and *Dolichotis patagonum*), who removed more seeds than the seed predator in the FR. The FR shows higher habitat homogeneity than the UFR, and functional groups respond differently to habitat heterogeneity at different spatial scales. In the FR, because large herbivores are locally extinct (e.g. *Lama guanicoe*) and domestic herbivores are excluded, important functions of large herbivores are missing, such as the maintenance of habitat heterogeneity, which provides habitats for medium-sized opportunistic frugivores with consequent improvement of quality and quantity of seed dispersal services. In the UFR, with low densities of large herbivores, probably one important ecosystem function this group performs is to increase habitat heterogeneity, allowing for the activity of medium-sized mammals who, behaving as opportunistic frugivores, did the most significant seed removal.

## Introduction

Among the ecological functions performed by mammals, the seed dispersal can be considered an ecosystem service that controls the long-term dynamics of plant communities and the recovery of vegetation in human-disturbed habitats [1], and contributes to human well-being through the regulation of ecosystem processes [2]. This ecological function depends on species interactions, a key component of biodiversity that can be affected by anthropogenic drivers, in some cases even before species loss occurs [3].

In drylands, where natural variations in climate, topography, soil and vegetation result in a high habitat heterogeneity [4,5], the land use histories involving different management strategies bring about complex changes in vegetation composition and habitat structure that lead to changes in richness, abundance, and functional diversity of mammals [6,7,8,9]. Moreover, we know almost nothing about the effects of human-induced disturbances on seed dispersal by mammals in dry ecosystems.

Some fruits of *Prosopis* species were considered to have traits involved in the megafaunal dispersal syndrome, and they could be viewed as anachronisms [10]. The large and indehiscent fruits containing sugar, oil, or nitrogen rich pulp, the seeds protected by a thick, tough or hard endocarp or seed coat that usually allows seeds to pass intact by the molars and through the digestive tract when eaten by large mammals, are some of the traits molded by evolutionary interactions with the extinct Pleistocene megafauna [10]. Currently, the drylands where *Prosopis* forests occur in Argentina are suffering the loss or local extinction of native herbivores (e.g. *Lama guanicoe*, *Rhea americana*, *R*. *tarapacensis*), a new pulse of animal loss that was globally described as the Anthropocene defaunation [11]. Given this scenario, the seed dispersal function could be changing since the large native mammals are all under threat or have decreasing populations, which could be compensated for by some small and medium-sized species [8] and domestic animals who behave as alternative dispersers [12,13].

Traditionally, conservation efforts to promote the protection of ecosystems against disturbances include as the main approach the change in the primary land-use by establishing protected areas, either strictly protected or less strictly protected [14,15], sometimes using fencing as a tool for conservation purposes. With costs and benefits, fences can protect biodiversity by excluding threatening processes, such as grazing by domestic herbivores [16] but there is a need to better understand how the delivery of ecosystem services (e.g. seed dispersal by animals) is compromised by fencing initiatives [17]. For example, in the Monte Desert of Argentina, the Man and Biosphere Ñacuñán Reserve was created with the goal to protect *P*. *flexuosa* woodlands using as intervention the exclusion of grazing by fencing and the passive recovery of vegetation [18]. Nevertheless, considering that large native herbivores are locally extinct in some areas of the Monte, it is not easy to predict the effect of excluding domestic herbivores involved in the *P*. *flexuosa* seed dispersal process because these animals, as opportunistic frugivores, play the role of seed dispersers through endozoochory [12,13,19,20]. Added to this, through trampling, reduction of vegetative cover, and changes in the quantity and quality of available food, large herbivores cause modifications in ecosystem functioning and habitat heterogeneity at different spatial scales. Faced with these changes, medium and small-sized mammals involved in seed dispersal respond differentially and they could be favored or disadvantaged [21,22].

When viewed from a functional perspective, conservation through management interventions applied to protected areas should aim to ensure that the occurring species are the ones maintaining the desired ecosystem properties within acceptable bounds. Unfortunately, there are still very few examples of empirical assessment of ecosystem functions in drylands under different management interventions. This study focused on *P*. *flexuosa* seed dispersal by mammals, using seed removal and number of animal visits as an estimator of the quantitative component of seed dispersal [23,24] performed by different functional mammal groups. The study was carried out in protected areas under two management interventions: a fenced reserve with grazing exclusion and absence of large native mammals (Ñacuñán Reserve; FR) and an unfenced reserve where there are low densities of large native and domestic animals (Ischigualasto Park; UFR). The objectives were: 1. to assess the relative contributions to seed removal by different functional mammal groups under the two interventions; 2. to analyze habitat heterogeneity at different spatial scales under the two interventions; 3. to evaluate how seed removal by different functional groups responds to habitat heterogeneity at different spatial scales.

## Materials and Methods

### Study area

The Monte Desert occupies approximately 460 000 km ^2^ of the dry Argentinian west [25]. Climatically, it is an arid to semiarid region, with mean annual precipitation ranging from 30 to 350 mm, and temperatures ranging from a mean maximum of 25.2°C and a mean minimum of 10.2°C in its northern part, to 20.4 and 7.3°C in the southern portion [26]. The three most extensive natural areas are the Northern (25–30°S), Central (30–37°S) and Southern Monte (37–43°S) according to the endemic assemblages [26]. From an economic point of view, the most important plant community in the Northern and Central Monte regions is the open woodland of *P*. *flexuosa* (“algarrobal”). Historically, *P*. *flexuosa* wood was used for firewood, charcoal, post for training vines, and as timber for furniture and flooring. *Prosopis flexuosa* fruits (pods) are used as food resources by rural populations and domestic animals. The most common disturbances affecting woodlands have been deforestation, grazing and fires in the non-irrigated areas and the replacement of natural ecosystems by croplands in irrigated oasis [27]. The effects of the past use of *P*. *flexuosa* woodlands are difficult to revert because of this species' low rate of regeneration and growth. This low rate of regeneration would be related to the temporal variability of seed production, to seed and seedling predation, and to the low frequency of occurrence of the climatic conditions promoting its establishment [27,28].

**Fig 1:**
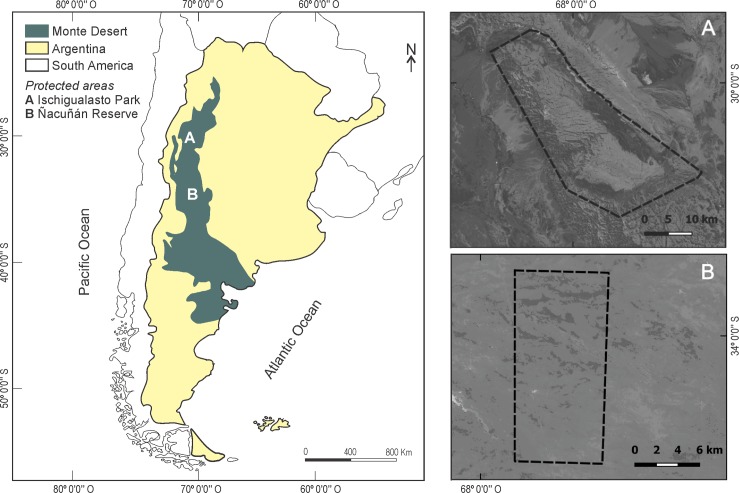
Location of the Monte Desert and the protected areas included in the study. (A) The Ischigualasto Provincial Park (UFR) and (B) the Man and Biosphere Ñacuñán Reserve (FR). The boundaries of the protected areas are indicated by dashed lines.

Because of the fast loss of forests, in Argentina native woodlands are currently protected by national and provincial laws [29] (National Law 26331). Some relict *P*. *flexuosa* woodlands are included in the two protected areas where this study was conducted: the Man and Biosphere Ñacuñán Reserve in the Central Monte and the Ischigualasto Provincial Park in the Northern Monte. The Ñacuñán Reserve (FR; 340° 02' S, 670° 58' W) is located in Mendoza Province (Fig 1) and it is the only fenced protected area in the Monte free from livestock since 1972. The reserve encompasses 12,800 ha and it has an average annual precipitation of 326 mm [18]. After a 50-year grazing exclusion, the passive recovery of the native vascular flora is remarkable [30]. Large native mammals, such as *Lama guanicoe*, which are known to disperse *P*. *flexuosa* seeds [20] have been present in the reserve in the past [18] but are now locally extinct. For the purposes of this work, the FR represents a site lacking large herbivores but supporting different functional mammal groups involved in the *P*. *flexuosa* seed dispersal process: medium-sized mammals acting as opportunistic frugivores that disperse seeds through endozoochory (the native species *Dolichotis patagonum*, *Lagostomus maximus*, and *Lycalopex griseus;* the exotic species *Lepus europaeus*) [19], scatter-hoarding seed dispersers (*Microcavia australis* and *Eligmodontia typus*) [13,31,32], and a larder-hoarding seed predator (*Graomys griseoflavus*) [31].

The Ischigualasto Park (29° 55' S, 68° 05' W) is located in San Juan Province (Fig 1) and it extends over 62,916 ha. The average annual precipitation in the area is 183 mm. The park protects the same seed-dispersing functional groups as the Ñacuñán Reserve. However, large herbivores are added to the group of opportunistic frugivores: the native species *L*. *guanicoe*, and low densities of exotic herbivores, such as *Equus asinus* (donkey), *E*. *ferus caballus* (horse), *Bos taurus* (cow), and *L*. *europaeus* [13,20,33]. The provincial administrations (the Direction of Conservation and Protected Areas of San Juan and the Direction of Renewable Natural Resources of Mendoza) fully authorized to conduct this research in the protected areas.

### Seed removal experiment

The experiment was conducted during the fruiting season in February 2015. Forty and 34 sampling stations, in the Ñacuñán Reserve and in the Ischigualasto Park respectively, were randomly chosen under *P*. *flexuosa* trees with similar crown diameter (approximately 5 m). The minimum pairwise distance between the trees was 500 m. At each sampling station, 20 *P*. *flexuosa* fruits containing in total 300 seeds, were offered for 48 h. The short fruit exposure time helped prevent fruit removal by ants (Campos C., pers. obs.). In order to identify animal species and quantify the number of seeds removed by each of them, we placed a camera trap (Moultrie 990i) at 1.80 m height focused on each sampling station. Previously tested for their best set up, the cameras took three pictures whenever animal movement was detected, with a 30-second delay between consecutive pictures. The set up and the location of the cameras allowed us not only to identify species, but also to count the number of seeds removed by animals and to quantify the number of visits removing seeds [34]. Removed seeds were those that were cached and moved away from the camera trap coverage by an animal.

Considering functional groups as sets of species showing similar effects on major ecosystem processes [35], the following functional groups were defined based on previous studies [13,19,20,31,32]: “opportunistic frugivores”, “scatter-hoarders”, and “seed predators”. Species with as yet unknown functional roles were grouped as “others”.

### Estimating vegetation structure from remote sensing data

Because in drylands, like the Monte Desert, plants have small leaf areas, non-photosynthetic tissues for long periods and are distributed in patches scattered throughout a matrix of bare soil [36], the most commonly used green index does not usually have a good fit. Therefore, the SATVI green index (Soil- Adjusted Total Vegetation Index) [37] was calculated which, being sensitive to both green and senescent vegetation and including a parameter that normalizes for the effect of bare soil, would be the best predictor of vegetation cover in the Monte [36,38]. This green index was the basis for image texture analyses conducted to assess vegetation heterogeneity. Images of the study areas used for the analyses were Landsat 8 OLI scenes (30-m resolution; path 231 and row 84 for Ñacuñán Reserve and path 232 and row 081 for Ischigualasto Park) acquired on 7 June 2015 and 29 May 2015 respectively (USGS EROS: http://eros.usgs.gov) were used. These date were selected because the images had a lower cloud cover (8.46% for Ñacuñán Reserve and 6.54% for Ischigualasto Park). Images were rescaled to the Top Of Atmosphere (TOA) reflectance with a correction for the sun angle using coefficients provided in the product metadata file (MTL file).

Image texture analysis is a remote sensing approach to spatial variability in gray level (i.e. gray shadow of pixels); hence, it contains important information about the spatial and structural arrangement of objects in an image [39,40]. First-order texture measures are based on the number of occurrences of each gray-level within a given processing window. Second-order texture measures use a gray-level spatial dependence matrix (i.e. gray-level co-occurrence matrix) to calculate texture values [39], which indicates the probability that each pair of pixel values co-occurs in a given direction and distance [39,40].

Several window sizes were evaluated, since vegetation structure at different scales may affect the presence of different functional mammal groups involved in seed removal. The different scales were represented by the extent of the moving window of an image texture measure, i.e. with 3 x 3, 5 x 5, 7 x 7 and 9 x 9 30-m pixel moving windows. The first-order texture measures on SATVI were calculated using the different sizes of moving windows, i.e. the pixel values within a moving window were used to calculate a statistic that was assigned to the central pixel [39]. Second-order texture measures were calculated on SATVI using the same moving windows, but the pixel values were first translated into a gray-level co-occurrence matrix (GLCM), which allowed us to consider the relationship among neighboring pixels [39]. Second-order texture measures were calculated in four directions, i.e. from the GLCM computed at 0° (horizontal neighbors), 45° (diagonally right), 90° (vertically), 135° (diagonally left), and averaged [39].

Some first-order texture measures are strongly correlated with second-order measures (i.e. mean, variance, and entropy) [41]; therefore, second-order measures were selected because they considered the spatial relationships of pixels. The following subset of texture measures was used: first-order (range) and second-order (mean, variance, contrast, entropy, and second moment). All texture measures were finally stored as separate layers in the GIS and were extracted for each sampling station. Quantum GIS [42] and ENVI GIS [43] were used in image analysis.

### Data analysis

To assess whether seed removal differs among functional mammal groups under the two interventions (FR and UFR), a generalized linear mixed model (GLMM) with a Poisson error structure [44] was fitted for number of seeds removed as the response variable. The interaction between functional groups (with four levels: “opportunistic frugivores”, “scatter-hoarder”, and “seed predator”) and interventions (FR and UFR) were included as explanatory variables, and the number of visits was considered an offset. Trees were considered a random factor. However, because model exhibited overdispersion (*ĉ* = 9.56), a negative binomial distribution was finally adjusted. The “others” functional group was excluded from the model because seed removal by this groups was very low.

To analyze the habitat heterogeneity under the two interventions, redundancy analyses (RDAs) were applied at every spatial scales to the matrix of mean, variance, contrast, entropy, second moment, and range (response variables) as an overall measure of relationship between the two sets of variables, corresponding to the interventions (FR and UFR), used as nominal explanatory variables. This constrained ordination assess association based on similarity and was performed because data sets had a short gradient (L = 2.69) indicating linear response curves, and explanatory variables were in the form of categorical predictors [45]. Conditional effects of explanatory data on habitat data were assessed using Monte Carlo permutation test (199 randomizations) and the percentage of the explained variability was used as a measure of explanatory power.

To evaluate how seed removal by each functional group responds to habitat heterogeneity at different spatial scales, GLMMs with a Poisson error structure [44] were fitted for each scale considered (i.e. 3 x 3, 5 x 5, 7 x 7 and 9 x 9 30-m). Poisson errors are widely used in the analyses of count data, but in these analyses they resulted in highly overdispersed and zero-inflated models [46], making the zero-inflated negative binomial a more adequate error structure. The response variable for models was the number of seeds removed by each functional group; the type of intervention (FR and UFR) was included as a random effect and the number of visits was an offset. The texture measures that were not correlated were included as explanatory variables. Spearman rank correlation, a non-parametric measure of statistical dependence [47], was used to identify collinearity between independent variables. It is important to identify high collinearity because it can result in coefficient estimates that are difficult to interpret as independent effects or have high standard errors [48]. Because variables with the coefficient r > |0.8| were excluded, models had different fixed variables at each scale, i.e. mean, variance and entropy for 3 x 3 scale, and only mean and variance texture measures for the other scales. A backward elimination procedure was performed to remove insignificant terms without losing important information. Backward elimination started with all of the predictors in a full model. The least significant variable, i.e. the one with the largest *P* value, was removed and the model was refitted. Each subsequent step removed the least significant variable in the model until all remaining variables had individual *P* values less than 0.05. The sign of parameters having significant effects was used to interpret the results [49,50]. Correlograms with the Pearson residuals of each best model were fitted to check for spatial-autocorrelation among sampling points [48]. Evidence of spatial dependence affecting the models was not detected.

All statistical analyses and graphs were performed using R 3.2.2 language and environment [51]. Different packages were used: “vegan” package [52] for RDA analysis; “glmmADMB” package [53,54] for model building and “ncf” for spatial auto-correlation [55].

## Results

### Seed removal

During the 148 nights-camera traps (80 for FR and 68 for UFR), 70% of offered seeds were removed only by mammals (32% in FR and 38% in UFR). Seven species were recorded removing *P*. *flexuosa* seeds and they were classified according to their functional roles (Table 1).

**Table 1:** Mammal species removing *P*. *flexuosa* seeds in the two protected areas.

Animal species	Mean percentage of seeds removed ± SE	Functional groups
*Lycalopex griseus*	27.87 ± 4.63	opportunistic frugivores
*Graomys griseoflavus*	17.67 ± 3.81	seed predators
*Microcavia australis*	13 ± 3.34	scatter-hoarders
*Zaedyus pichiy*	4.67 ± 2.09	others
*Dolichotis patagonum*	4.33 ± 2.15	opportunistic frugivores
*Conepatus chinga*	1.33 ± 1.09	others
*Ctenomys mendocinus*	0.20 ± 0.20	others

Mean percentage of seed removed ± SE under 40 tress in the FR (Ñacuñán Reserve) and 34 trees in the UFR (Ischigualasto Park), and the functional group in which mammal species were included are indicated.

**Fig 2:**
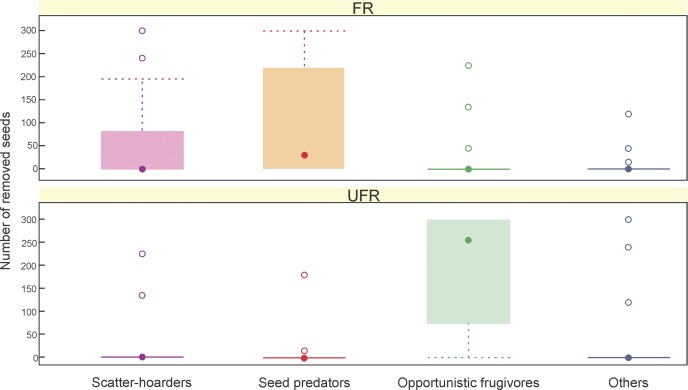
Boxplot of number of *P*. *flexuosa* seeds removed by the different functional mammal groups in the FR (Ñacuñán Reserve) and the UFR (Ischigualasto Park). The horizontal bold line in the box indicates the median value of the data. The upper and lower 1 hinges of the box indicate the 75th and the 25th percentiles of the data set, respectively. The ends of the vertical lines indicate the minimum and maximum data values; the points outside the ends of the whiskers are outliers.

The most important functional groups of seed removers were the seed predator and the scatter-hoarder in the FR, and the opportunistic frugivores in the UFR (Fig 2). The model including the functional groups and interventions applied to the protected areas showed that the type of intervention (FR or UFR) and the interaction between intervention and functional groups explain seed removal by mammals. It was found that seed removal by opportunistic frugivores (*L*. *griseus* and *D*. *patagonum*) in the UFR is higher than seed removal by the seed predator (*G*. *griseoflavus*) in the FR (Table 2).

**Table 2:** Generalized linear mixed models (GLMMs) to evaluate how the different functional groups (seed predator, opportunistic frugivores, and scatter-hoarder) and interventions (FR: Ñacuñán Reserve and UFR: Ischigualasto Park) explained *P*. *flexuosa* seed removal.

	Parameter estimate ± SE	Z value	*P* value
intercept	0.85±0.68	1.25	0.21
UFR	-4.07±1.15	-3.53	0.00041***
opportunistic frugivores	-1.23±1.02	-1.21	0.23
scatter-hoarder	0.31±0.85	0.36	0.72
UFR: opportunistic frugivores	3.42±1.49	2.30	0.02*
UFR: scatter-hoarder	0.94±1.43	0.66	0.51

Parameter estimates (± SE), Z and *P* values for significance (*: *P* < 0.05, ***: *P* < 0.001).

### Habitat heterogeneity at different spatial scales under the two interventions

Regarding the analysis of habitat heterogeneity based on the RDA of habitat data at different spatial scales, a correlation was found between habitat data and the explanatory variable at every spatial scale for the first three axes, which cumulatively explained more than 90% of the variance (Table 3). The FR was closely associated with the second moment, showing a more homogeneous distribution of vegetation at every scale in comparison with the UFR. Moreover, the UFR was more closely related to mean, variance, contrast, and entropy of SATVI, showing a high coverage and heterogeneity of vegetation across every spatial scale.

**Table 3:** Results of redundancy analysis (RDA) of habitat data (mean, variance, contrast, entropy, second moment, and range) at different spatial scales using the two interventions (FR: Ñacuñán Reserve and UFR: Ischigualasto Park) as nominal explanatory variables.

Spatial scale	R^2^	F	*P* value	first axis (explanation of total variability)	second axis	third axis
3 x 3	0.42	52.37	0.005	0.42	0.34	0.21
5 x 5	0.47	64.62	0.005	0.47	0.31	0.17
7 x 7	0.48	65.57	0.005	0.48	0.33	0.12
9 x 9	0.46	60.41	0.005	0.46	0.37	0.10

### Response to habitat heterogeneity by the functional mammal groups

When considering habitat heterogeneity from texture measures on SATVI, the best model to explain removal by the scatter hoarder (*M*. *australis*) included mean at 5 x 5, and variance and mean at 7 x 7 and 9 x 9 spatial scale, i.e. the number of seeds removed by the scatter-hoarder increased with decreasing mean and increasing variance of SATVI (Table 4). For the seed predator (*G*. *griseoflavus*), the best model to explain seed removal included mean at 3 x 3 and 5 x 5 spatial scales, i.e. seed removal increased when the mean decreased. At higher scales (7 x 7 and 9 x 9) seed removal by the seed predator increased when variance decreased (Table 4). For the opportunistic frugivores (*L*. *griseus* and *D*. *patagonum*), the best model included mean and entropy at 3 x 3, and variance at 5 x 5 spatial scale, i.e. the removal of seeds by frugivores increased with increasing mean and entropy, and decreasing variance (Table 4). For the “others” functional group, the adjusted models included entropy at 3 x 3 spatial scale, variance at 5 x 5, and mean and variance at 7 x 7, i.e. seed removal by this functional group increased when entropy increased at 3 x 3, variance decreased at 5 x 5, and mean and variance increased at 7 x 7 spatial scale (Table 4).

**Table 4:** Generalized linear mixed models (GLMMs) to assess how the different functional groups (scatter-hoarder, seed predator, opportunistic frugivores, and others) respond to habitat heterogeneity at different spatial scales (3 x 3, 5 x 5, 7 x 7 and 9 x 9 30-m pixel moving windows).

		Functional groups											
Texture measure		Scatter-hoarder			Seed predator			Opportunistic frugivores			Others		
		Parameter estimate ± SE	Z	*P*	Parameter estimate ± SE	Z	*P*	Parameter estimate ± SE	Z	*P*	Parameter estimate ± SE	Z	*P*
3 x 3													
	intercept	-1.57±3.93	-0.40	0.69	10.07±2.86	3.52	***	-51.7±6.57	-7.87	***	3.29±9.03	0.36	0.72
	mean		-0.39	0.70	-0.29±0.08	-3.42	***	0.51±0.11	4.77	***			
	variance	-0.74±0.75	-0.99	0.32				-0.38±0.45	-0.86	0.39			
	entropy	3.12±2.13	1.46	0.14				16.3±2.79	-3.05	***	6.49±0.37	17.4	***
5 x 5													
	intercept	11.4±3.49	3.25	**	9.82±2.93	3.36	***	-3.02±6.01	-0.50	0.62	0.06±3.10	0.02	0.99
	mean	-0.33±0.11	-3.10	**	-0.28±0.09	-3.24	**				0.11±0.09	1.19	0.23
	variance	0.39±0.27	1.46	0.14				-0.53±0.26	-2.06	*	-1.20±0.21	-5.63	***
7 x 7													
	intercept	11.9±2.89	4.11	***	1.35±0.42	3.20	**	7.73±8.07	0.96	0.34	-31.2±3.02	-10.33	***
	mean	-0.34±0.09	-3.74	***				-0.22±0.26	-0.84	0.40	0.69±0.10	6.85	***
	variance	0.56±0.17	3.37	***	-0.40±0.11	-3.72	***	0.12±0.26	0.48	0.63	0.53±0.14	3.74	***
9 x 9													
	intercept	11.6±2.88	4.02	***	1.32±0.42	3.15	**	3.87±9.29	0.42	0.68	-543±6751	-0.08	0.94
	mean	-0.33±0.09	-3.65	***				-0.25±0.27	-0.94	0.35	10.5±127.4	0.08	0.93
	variance	0.50±0.16	3.20	**	-0.35±0.09	-3.60	***	0.23±0.15	1.50	0.13	16.4±218	0.08	0.94

Parameter estimates (± SE), Z and *P* values for significance (*: *P* < 0.05, **: *P* < 0.01, ***: *P* < 0.001).

## Discussion

As an approach to the quantitative component of seed dispersal process, the results are showing that seed removal by different functional mammal groups can be affected by the interventions applied to protected areas. In the FR, the most important seed removers were a seed predator and a scatter-hoarder, whereas the opportunistic frugivores were the major removers in the UFR were. The FR shows more habitat homogeneity than the UFR, and the functional groups respond differently to habitat heterogeneity and to plant cover at different spatial scales.

Nearly all existing studies addressing the effects of human activities on seed dispersal by mammals come from tropical systems, where it was found that the relative importance of different seed-dispersing functional groups is changing because human-induced disturbances, such as logging, hunting, forest degradation, and fragmentation, are causing loss of species functionality due to selective local extinction mostly of large-sized dispersers [56,57].

Fruit and seed removal is the net outcome of animal activity, which may or may not result in seed dispersal away from the parent plant. Removal could lead to successful seed dispersal if done by seed dispersers (e.g. frugivores and scatter-hoarders), or to seed loss if done by seed predators, depending largely on animal feeding behavior, fruit processing, and post-feeding movements [58]. In response to our first objective, and considering the wide spectrum of *P*. *flexuosa* seed dispersers in the Monte Desert [13,33], we found that seed removal is accounted for different functional mammal groups under the two interventions. On the one hand, in the absence of large herbivores (FR), a seed predator (*G*. *griseoflavus*) and a scatter-hoarder (*M*. *australis*) were the major seed removers. These species are small-mammals (less than 2 kg) that consume fruits and seeds of *P*. *flexuosa* (seed predator) or only fruits (scatter-hoarder) [13,31,32]. Several studies show that areas with low abundance of large herbivores support high abundance of small rodents [59,60], and the ecological consequences of this trend in seed dispersal will depend on the species involved and their seed-dispersing role. In some cases, the functional extinction of a large seed predator (such as the white-lipped peccary *Tayassu pecari*) leads to the overcompensation of seed predation by small rodents, such as occurs in defaunated areas in the Atlantic forests [61]. In other cases, small rodents that disperse seeds by scatter-hoarding are the ones that actually compensate for the reduction in the abundance of large seed-dispersing mammals [62]. In our case, both a seed predator and a scatter-hoarder made the main contributions to fruit removal in the FR where large mammals are excluded or locally extinct.

On the other hand, in the UFR, despite large exotic and native herbivores are present and *P*. *flexuosa* seeds were found in their feces in previous studies (e.g. *L*. *guanicoe*, *Rhea americana*, cow, horse, donkey) [20,33], *L*. *griseus* and *D*. *patagonum* were the main seed removers. These two medium-sized species disperse *P*. *flexuosa* seeds by endozoochory. *Prosopis flexuosa* seeds found in feces of *L*. *griseus* and *D*. *patagonum* maintain 60–70% of viability, with the additional benefit that the passage through the digestive tract of dispersers kills 50% of bruchid larvae that parasitize seeds [13,19,20]. Both species could be considered long-distance seed dispersers; foxes (e.g. *Pseudalopex culpaeus*) travel daily distances that fluctuate between 6 to 8 km [63] whereas *D*. *patagonum* home ranges are between 33.25 and 197.5 ha [64].

The global reduction in range and abundance of large native herbivores, rendering them functionally extinct, or their replacement by livestock in much of their historic ranges, affect landscape structure and ecosystem functioning [65,66]. In relation to landscape structure, studies indicate that removal and extinction of large herbivores change vegetation composition and structure causing habitat homogeneity and creating less open landscapes [65,67]. Consistently with this, our analysis of habitat heterogeneity at different spatial scales under the two interventions shows that in the FR, where livestock was excluded and large native mammals are locally extinct, the distribution of vegetation turns out to be more homogeneous at every spatial scale in comparison with the UFR, where domestic and large native animals are present in low densities. In the FR, the habitat heterogeneity that existed previous to livestock exclusion tended to diminish, mainly due to colonization, distribution and expansion of some plant species over time, inducing spatial homogenization [27].

The reduction in habitat heterogeneity and in the quality and quantity of seed dispersal services are among the most likely impacts of large herbivore loss [67]. Habitat heterogeneity provides animals with a variety of refuges against predators, food resources, as well as diverse types of layers that affect their locomotion [68]. Animals use specific habitat patches, vegetation layers or cover classes according to their ecological requirements and, when habitat heterogeneity changes at different scales, the response of each species might be variable [69,70]. Image texture measures, which are a surrogate for vegetation structure, are useful for characterizing differences in habitat heterogeneity. They range from fine- to coarse-grained and therefore provide a combination of attributes that are desirable for characterization of wildlife habitat [71,41,72–74]. Wood and colleagues [41] found that variance applied to the vegetation index captured the variation in foliage-height diversity and horizontal vegetation structure in savannas. When the manner in which seed removal by different functional groups responds to habitat heterogeneity at different spatial scales was assesssed, it was found that the number of seeds removed by the scatter-hoarder (*M*. *australis*) at two larger scales was directly related to the variance in SATVI. This measure represents an estimation of the vegetation spatial heterogeneity [75], thus the scatter-hoarder removed more seeds in heterogeneous habitats, and in habitats with low plant cover at scales 5 x 5 and 7 x 7. In the Monte Desert, burrows of *M*. *australis* reach their highest density in the mesquite community, under *P*. *chilensis* or *P*. *flexuosa* trees [76,77] and mainly occur in grazing lands where the landscape is heterogeneous [78].

For the seed predator group, represented by *G*. *griseoflavus*, a tendency was found towards high seed removal in homogeneous habitats (at 7 x 7 and 9 x 9), represented by a low variance in SATVI, which is in keeping with the tendency to avoid complex landscape units in the FR found for this species by Tabeni and coauthors [79]. At small spatial scales (3 x 3 and 5 x 5), the seed predator removed more seeds in habitats with low plant cover. But at microhabitat scale (2 x 2 m) it was found that *G*. *griseoflavus* selected high cover of litter and subshrubs in the mesquite forest, because plant cover provides safe places to avoid predation [80].

Seed removal by the opportunistic frugivores was higher when vegetation cover increased at the lower scale considered (3 x 3), according to the mean of SATVI, and low habitat heterogeneity at the 5 x 5 scale. Previous studies found that, across its geographical range in desert areas, *D*. *patagonum* selects open scrubland, sparsely vegetated and with a high proportion of bare soil [64,78,81]. It was suggested that this species might benefit from open habitats, where its predator detection efficiency increases [82]. In the case of *L*. *griseus*, it occurs in a variety of habitats but prefers shrubby open areas [83]. However, there are not studies on *D*. *patagonum* and *L*. *griseus* assessing habitat selection at the scale considered in our research. Probably, the habitat selected by these species is mainly open at broad scale but they require high vegetation cover at lower scales, though this assumption deserves future research.

Finally, the “others” functional group, composed of *Z*. *pichiy*, *C*. *mendocinus*, and *C*. *chinga*, removed more *P*. *flexuosa* seeds in homogeneous habitats at 5 x 5, and in habitats with high plant cover and heterogeneity at 7 x 7 spatial scale. Almost nothing is known about the habitat requirements of these species in the Monte Desert and, although they removed few seeds, this is the first study to record their contribution to *P*. *flexuosa* seed removal.

In summary, using camera traps to identify species removing *P*. *flexuosa* seeds and image texture as a tool for estimating habitat heterogeneity (i.e. structure of vegetation), this study considered different functional groups' responses to management interventions in protected areas of Argentinian drylands. In the UFR, with low densities of large herbivores, probably the most important ecosystem function performed by this group is related to their increase of habitat heterogeneity, allowing for the activity of medium-sized mammals, who made the most significant seed removal. Although many aspects associated with quality of seed dispersal by *D*. *patagonum* and *L*. *griseus* are still unknown, endozoochory by these species is known to have some advantages for *P*. *flexuosa* seeds [13,19,20].

In the FR, the functional group of opportunistic frugivores appears to be disadvantaged. In this reserve, where cows and horses are totally excluded and the large native herbivore *L*. *guanicoe* is locally extinct, the medium-sized mammals actually do meet their habitat requirements in more heterogeneous habitats outside the protected area [69,78]. And even though the scatter-hoarder (*M*. *australis*) does remove seeds, the seed predator (*G*. *griseoflavus*) appears to be as important a seed remover as the scatter-hoarder. In the FR, two important ecological functions of large herbivores are missing: maintenance of habitat heterogeneity and improvement of quality and quantity of seed dispersal services. In this way, the local loss of large herbivores has direct and indirect effects on *P*. *flexuosa* seed dispersal: a decline in the provision of habitats for medium-sized opportunistic frugivores and the loss of seed dispersal by large herbivores themselves. It should be noted that the opportunistic frugivores have higher potential to disperse seeds longer distances than the scatter-hoarder group, an important fact considering the importance of long-distance seed dispersal in past and ongoing range expansions and colonisation processes of plant species facing the rapidly changing climate [84].

Future conservation management plans for protected areas could rely on the functional diversity rather than on random species conservation, considering that the extinct megafauna was responsible for maintaining a more open and varied landscape in the past. Thus, inside the FR, for example, clearing areas adjacent to fences and dirty roads, and maintaining tree structure could enable the activity and movement of *P*. *flexuosa* seed dispersers, such as scatter-hoarders and opportunistic frugivores, who will be able to access fruiting trees and remove fruits and seeds. In the UFR reserve, continuous monitoring of abundance of exotic large species would be needed in order to prevent overgrazing and the consequent impact on the ecosystem.

## Supporting Information

S1 Table Dataset of texture measures and seed removal by the functional mammal groups in UFR and FR. (XLS)
